# Microbial community structure and shift pattern of industry brine after a long-term static storage in closed tank

**DOI:** 10.3389/fmicb.2022.975271

**Published:** 2022-09-02

**Authors:** Demei Tu, Juntao Ke, Yuqing Luo, Tao Hong, Siqi Sun, Jing Han, Shaoxing Chen

**Affiliations:** ^1^College of Life Sciences, Anhui Normal University, Wuhu, China; ^2^Anhui Jiaotianxiang Biological Technology Co., Ltd., Xuancheng, China; ^3^State Key Laboratory of Microbial Resources, Institute of Microbiology, Chinese Academy of Sciences, Beijing, China

**Keywords:** salt mine, hypersaline environment, halophiles, haloarchaea, microbial community, archaea

## Abstract

Brine from Dingyuan Salt Mine (Anhui, China), an athalassohaline hypersaline environment formed in the early tertiary Oligocene, is used to produce table salt for hundreds of millions of people. However, halophiles preserved in this niche during deposition are still unknown. Here, we employed cultivation and high-throughput sequencing strategies to uncover the microbial community and its shift after a long-term storage in the brine collected from Dingyuan Salt Mine. High-throughput sequencing showed (1) in the fresh brine (2021), *Cyanobium_stocktickerPCC-6307* spp. (8.46%), *Aeromonas* spp. (6.91%) and *Pseudomonas* spp. (4.71%) are the dominant species in bacteria while *Natronomonas* spp. (18.89%), *Halapricum* spp. (13.73%), and *Halomicrobium* spp. (12.35%) in archaea; (2) after a 3-year-storage, *Salinibacter* spp. (30.01%) and *Alcanivorax* spp. (14.96%) surpassed *Cyanobium_stocktickerPCC-6307* spp. (8.46%) becoming the dominant species in bacteria; *Natronomonas* spp. are still the dominant species, while *Halorientalis* spp. (14.80%) outnumbered *Halapricum* spp. becoming the dominant species in archaea; (3) *Alcanivorax* spp. and *Halorientalis* spp. two hydrocarbons degrading microorganisms were enriched in the brine containing hydrocarbons. Cultivation using hypersaline nutrient medium (20% NaCl) combined with high-throughput 16S rRNA gene sequencing showed that (1) the biomass significantly increased while the species diversity sharply declined after a 3-year-storage; (2) *Halorubrum* spp. scarcely detected from the environment total stocktickerDNA were flourishing after cultivation using AS-168 or NOM medium; (3) twelve possible new species were revealed based on almost full-length 16S rRNA gene sequence similarity search. This study generally uncovered the microbial community and the dominant halophiles in this inland athalassohaline salt mine, and provided a new insight on the shift pattern of dominant halophiles during a long-term storage, which illustrated the shaping of microorganisms in the unique environment, and the adaptation of microbe to the specific environment.

## Introduction

Hypersaline ecosystems are widespread across the globe, including a wide variety of habitats such as hypersaline lakes, solar salterns, soils, and ancient salt deposits (Oren, 2011). In addition to high salt concentrations, high pH, and low oxygen concentrations are also characteristics of these extreme environments (Naghoni et al., 2017). Although the conditions are harsh, and even high salinity is fatal to most organisms, a large number of halophilic archaea, halophilic and salt-tolerant bacteria still exist in these environments (Oren, 2002; Fendrihan et al., 2006; Naghoni et al., 2017), which play a vital role in global biogeochemical cycles (Mani et al., 2012).

In recent years, the diversity of microorganisms in various hypersaline environments has been studied, but these studies have mainly focused on saline lakes, solar salterns, saline soils (Ben Abdallah et al., 2018; Gomez-Villegas et al., 2018; Bachran et al., 2019; Couto-Rodríguez and Montalvo-Rodríguez, 2019; Nan et al., 2020; Sáenz de Miera et al., 2021). It is worth noting that salt mine, a unique habitat formed tens of millions of years ago, is also an important representative of the hypersaline environment (Chen et al., 2019). For instance, brine from Dingyuan Salt Mine (Dongxing Town, Dingyuan County, Anhui Province, China), an athalassohaline hypersaline environment, is used to produce table salt for hundreds of millions of people. However, current analysis of microbial communities in this kind of environment is limited. Actually, salt mines are rich in microbial resources. For example, the study of halophilic microorganisms in salt mines can not only reveal the evolution and adaptation mechanism of life in extreme environments, but also shows importance in the breeding, development and utilization of microorganisms (Chen et al., 2019).

At present, high-throughput sequencing technology has been applied to investigate microbial communities in different hypersaline environments (Genderjahn et al., 2018; Perez-Fernandez et al., 2019; Zhu et al., 2020). Compared to traditional methods, it has emerged as a reliable tool for investigating differences in microbial community species diversity and structure in any given habitat (Boutaiba et al., 2011), because the culture-independent method of amplicon sequencing appears to be more efficient than the traditional culture-dependent methods (Gibtan et al., 2017). However, culture-dependent method is still a necessary means to acquire valuable microbial strains with potential for new applications and to understand their ecophysiological and environmental functions (Vandamme et al., 1996; Sfanos et al., 2005). For example, the use of traditional culture techniques and the development of new media are encouraged to find novel pure isolates with desired physiological and metabolic characteristics (Rohban et al., 2009).

Therefore, in this study, high-throughput sequencing, clone library and traditional culture-dependent approaches were combined to analyze the microbial diversity of brine (long-term indoor sealed static storage and freshly collected brines) from Dingyuan Salt Mine in Anhui Province (China). The microbial community composition and diversity in this environment were explored using culture-independent and culture-dependent methods, and different results for characterizing microbial communities were compared. In addition, microbial interactions and keystone taxa in complex environments were identified and inferred using co-occurrence network analysis method. The results of this study expand our knowledge on microbial ecology in hypersaline environments.

## Materials and methods

### Sampling site description and sample collection

**FIGURE 1:**
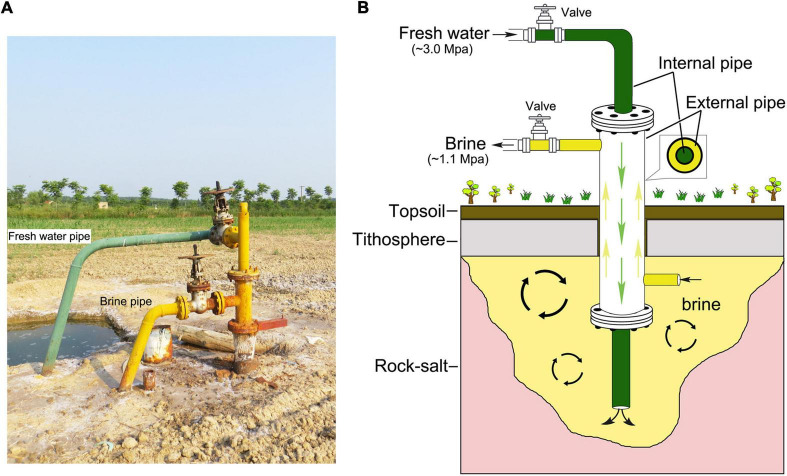
Landscape of the sampling site and brine production mode. **(A)** The surrounding landscape of the sampling site. The fresh water (green pipe) with high pressure was pumped into the underground salt mine and the industry brine (yellow pipe) with relative low pressure flowed out. **(B)** The working principle of rock salt mining is fresh water-in (green, ∼3.0 Mpa) and brine-out (yellow, ∼1.1 Mpa). Green arrows indicate the direction of fresh water in the internal pipe, and the yellow arrows indicate the direction of industry brine in the external pipe.

Brine samples were collected from Dingyuan Salt Mine located in Dongxing town, Dingyuan county, Anhui Province, China (117.4956E, 32.5066N) (Supplementary Figures 1a,b). Geographic location of the sampling site was generated with ArcGIS 10.2 software^1^. This sampling site is an athalassohaline hypersaline environment derived from a long-term evaporation and sedimentation of inland saline streams and lakes (Chen et al., 2019). Two brine samples, C4 and C5, were collected in August 2018, and the other three samples, C1, C2, and C3, were collected in July 2021. All these five samples were collected from the same site as shown in Supplementary Figures 1a,b and Figure 1A. And these brine samples were held using sterile plastic containers (10 L), and were brought back to the laboratory within a few hours. In particular, the two samples (C4 and C5) were stored in sealed plastic tanks for approximately 3 years in our laboratory without any manual intervention at perennial room temperature (20–25°C). And one-fifth of the volume of air existed in the upper layer of the tanks.

### Physicochemical properties determination

The pH of these brine samples value was measured by a Delta 320 pH meter (Mettler-Toledo, Zurich, Switzerland). Additionally, chemical composition of them was performed by a commercial analytical laboratory based on standardized methods (Beijing Zhongkebaice Technology Service Co., Ltd., Beijing, China). Nitrate ion (NO_3_^–^) concentration was measured by ion-chromatography on an ICS-1500 (Dionex, Sunnyvale, CA, United States). The content of metallic elements such as magnesium (Mg), iron (Fe), and potassium (K) were determined by inductively coupled plasma atomic emission spectrometry (ICP-AES) using an OPTIMA 5300 DV spectrometer (Perkin Elmer, Norwalk, CT, United States).

### Cultivation, isolation, and identification of halophilic microorganisms

The brine samples were cultured in two different hypersaline media AS-168 and NOM (pH = 7.5) (Han et al., 2007; Cui et al., 2012). Besides, solid media were made by adding 15.0 g agar powder to each liter of liquid medium prior to autoclaving. Then, 200 μL of above brine samples were pipetted and spread onto AS-168 and NOM agar plates (three plates for each sample). Finally, all plates sealed with parafilm were put into a ziplock bag to keep moisture during a long-term cultivation. After 3–4 weeks cultivation at 37°C, numerous halophilic or salt tolerant microorganisms were obtained.

Colonies, developed from the above operation with different color, transparency and size, were sorted out. After successive streaking, pure cultures were subjected to identification. Polymerase chain reaction (PCR) amplification of the 16S rRNA gene was performed using universal primers F8/R1462 (Supplementary Table 1; Lizama et al., 2001) and 27F/1492R (Supplementary Table 1; Lane, 1991) for archaea and bacterial respectively.

The PCR products were sequenced using the corresponding PCR primers (Sangon Biotech, Shanghai, China) after agarose gel electrophoresis. The assembled almost full-length 16S rRNA gene sequences (>1,300 nt) were used as queries to match the public database using Basic Local Alignment Search Tool (BLAST)^2^. To determine the taxonomic position of these strains, 97% of 16S rRNA gene sequence similarity was taken as species boundary.

### Total DNA extraction, 16S rRNA gene amplification, and high-throughput DNA sequencing

To analyze the microbial community of culture-dependent approach, colonies grown on AS-168 and NOM agar plates were collected by washing with 20% (w/v) sterilized NaCl solution. With this approach, cells of C1, C2, and C3 washed from AS-168 plates were blended into a tube as one sample (C1-3-AS168). Samples C4-5-AS168 and C4-5-NOM were obtained by the same method. Total DNA of these three samples was extracted using TIANamp Bacteria DNA Kit in accordance with the instruction (TIANGEN, Beijing, China).

To explore the microbial community of culture-independent approach, several related environmental samples such as brines, fresh water used to produce brine, ddH_2_O used to dissolve DNA, and routine lab air containing microorganisms were involved in the process of total environmental DNA extraction. Each sample (1 L) was filtered through a 0.22 μm membrane filter (as for lab air sample, an equal filtrating time with ddH_2_O was used). Then, the filter membrane of each sample was cut into small pieces, from which the total environmental DNA was extracted using the DNeasy PowerSoil Pro Kit (QIAGEN, Dusseldorf, Germany) according to the manufacturer’s instructions. Although long-term storage enables the formation of endospore in bacteria, the DNA extraction kit used in this study was also able to isolate total DNA *via* bead-beating from bacterial endospores (Redweik et al., 2020). Thus, differences in microbial diversity in different brine samples can be well characterized. Subsequently, all the harvested DNA samples were detected using 1% agarose gel electrophoresis, and the purity and concentration were determined by a NanoDrop-2000 (Thermo Fisher Scientific, Waltham, MA, United States).

The V3-V4 regions of bacterial and archaeal 16S rRNA genes from samples C1∼C5 were amplified using primer pairs 338F/806R (468 nt) for Bacteria, and Arch349F/Arch806R (457 nt) for Archaea, respectively (Supplementary Table 1; Derakhshani et al., 2016). Then, the 16S rRNA gene amplicons were subjected to high-throughput DNA sequencing on the Illumina Novaseq 6000 platform in Biomarker Biotech Co., Ltd. (Beijing, China).

### Clone library construction

In order to reveal the alteration of haloarchaeal community after a 3-year indoor storage in the brine sample in species level, the primer pair F8/R1462 (Supplementary Table 1; Lizama et al., 2001) was used to amplify the nearly full length of haloarchaeal 16S rRNA gene. C1-3 was made by blending the total DNA of C1, C2 and C3 in identical volume; and the C4-5 was generated in the similar way. One microliter of C1-3 or C4-5 was used as template in PCR amplification. The 16S rRNA gene was purified using MonPur™ Gel & PCR Purification Kit (Monad, Shanghai, China) after DNA electrophoresis. The purified DNA fragments were inserted into pMD18T vector (TaKaRa, Tokyo, Japan) for DNA sequencing. Then, the nearly full-length 16S rRNA gene sequences were used to determine the taxonomic status *via* sequence similarity search against public database using BLAST as well.

### Data analysis

For the purpose of obtaining clean reads, Trimmomatic (version 0.33) (Bolger et al., 2014) software was used to filter the raw reads, and Cutadapt (version 1.9.1) (Martin, 2011) software was used to identify and remove primer sequences. Next, clean reads from each sample were spliced and filtered by using Usearch (version 10) (Edgar, 2010) software. Finally, effective reads for further analysis were obtained by removing the chimeras with software UCHIME (version 4.2) (Edgar et al., 2011). Operational taxonomic units (OTUs) were obtained by clustering effective reads at a similarity threshold of 97% (Stackebrandt and Goebel, 1994). Using SILVA as the reference database, the Naive Bayesina classifier was used to indicate the OTU’s taxonomic status (Quast et al., 2012). According to the OTU information, QIIME software was used to generate species abundance tables at different taxonomic levels (Caporaso et al., 2010). The R language tool was used to draw the community structure figures of the samples at each taxonomic level (R Core Team, 2013). The alpha diversity indexes of the samples were evaluated by using the QIIME2 software (Hall and Beiko, 2018) and the rarefaction curve were generated with the R language tool (R Core Team, 2013). One-way analysis of variance was performed using SPSS 23.0 (Kirkpatrick, 2015) for counting DNA concentration and Shannon index of different samples, and *post-hoc* Scheffe test was used for pairwise comparisons. The level of significance was set at 0.05. Independent samples *t*-test was performed using SPSS 23.0 (Kirkpatrick, 2015) for the inorganic ion concentrations of samples C1-3 and C4-5, the level of significance was set at 0.05. The results obtained by clone library and culture-dependent methods were counted, and the bioinformatics analysis images were drawn using the Origin software (Edwards, 2002). According to the abundance and changes of each species in each sample, Spearman’s rank correlation analysis was performed (Corder and Foreman, 2014). Then, the data with significant and robust correlations (ρ > 0.7 and *P* < 0.05) were screened, and the correlation network diagram was drawn based on Python.

## Results

### Physicochemical characteristics of brine samples

Brine samples were collected from Dingyuan Salt Mine located in Dongxing Town, Dingyuan County, Anhui Province, China (Supplementary Figures 1a,b). The physicochemical properties of these brine samples are shown in Table 1. The pH of brine samples varies from 7.99 to 8.19, indicating that these brine samples are slightly alkaline. The concentration of nitrate ion in these five brine samples ranges from 8.1 to 9.8 mg/L. In addition, the concentrations of magnesium, potassium, and iron in samples C1-3 and C4-5 were not significantly different (*p* < 0.05).

**TABLE 1:** Physicochemical properties of brines.

Item	C1^a^	C2^a^	C3^a^	C4^b^	C5^b^
Mg (mg/L)	7.452	7.610	7.011	7.112	7.031
K (mg/L)	213.5	221.2	211.9	210.8	215.4
Fe (mg/L)	0.222	0.267	0.151	0.236	0.214
NO_3_^–^ (mg/L)	9.790	9.716	8.201	9.625	8.096
pH	8.09	8.11	7.99	8.19	8.17

^a^Brine collected in July, 2021.

^b^Brine collected in August, 2018.

### Total environmental DNA extracted from different brine samples

**FIGURE 2:**
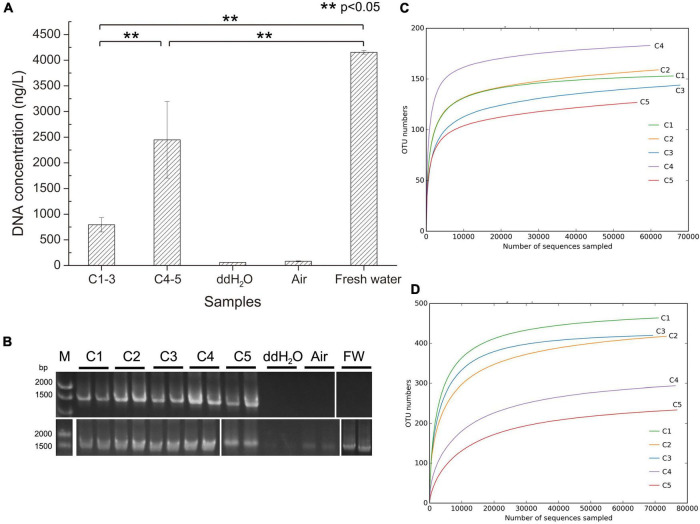
Total environmental DNA extraction and 16S RNA amplicon sequencing rarefaction curves. **(A)** The total DNA was extracted from 1 L of the corresponding samples except from the air in the lab with suction filtration approach. C1-3, three brine samples (C1, C2, and C3) collected in July 2021; C4-5, two brine samples (C4 and C5) collected in August 2018; ddH_2_O, distilled water; Air, time duration of lab air suction filtration was the same as ddH_2_O; Fresh water, the natural water source used to dissolve salt mines for brine production. **p < 0.05, significant difference. **(B)** These extracted environmental DNA was used as PCR template for amplifying haloarchaeal (upper row) and bacterial (bottom row) 16S rRNA gene using F8/R1462 and 27F/1492R, respectively. FW, fresh water; DNA ladder was shown on the left. **(C)** Rarefaction curve of archaeal 16S RNA amplicon sequencing. **(D)** Rarefaction curve of bacterial 16S RNA amplicon sequencing. OTU, operational taxonomic unit.

Total environmental DNA extracted from different brine samples is shown in Supplementary Table 2 and Figure 2A. The results revealed that the environmental DNA extracted from the samples collected in 2018 (C4-5) was 2–3 times higher than that from samples collected in 2021 (C1-3), which indicates that the biomass accumulated greatly after 3 years of indoor static and closed storage. The DNAs in the ddH_2_O used in environmental DNA extraction and room air samples were much lower than those in the brine samples in order of magnitudes. The DNA concentrations in the different samples (Figure 2A) were significantly different, demonstrating that there was a significant difference in DNA concentrations between the samples C4-5 and samples C1-3. Similarly, DNA concentrations in samples (C1-5) were all significantly different from sample freshwater (FW) which was pumped in to dissolve solid salt mine to produce brine. It was worth noting that the DNA concentration of the sample FW was much higher than that of the brine samples (C1-5). Additionally, the PCR amplification experiments indicated that no haloarchaeal DNA was detected or the amount of haloarchaeal DNA was below the limit of PCR detection in the samples ddH_2_O, room air and FW (Figure 2B), illustrating a low experimental contamination.

### Species diversity indices

High-throughput sequencing was performed on brine samples (C1-5), fresh water (FW), and samples collected after the halophiles in brine were cultured in medium (Cx-168 and Cx-NOM). Sequences obtained from the quality filtering were trimmed, after which the high-quality ones were obtained for further analysis. In the culture-independent approach, 398,634 and 399,340 high-quality reads were obtained with archaeal and bacterial 16S rRNA gene primer sets, respectively. For samples collected from culture-dependent of brine, 366,127 high-quality reads were obtained with archaeal 16S rRNA gene primer set. Interestingly, the number of OTUs of archaea in samples C1-3 and C4-5 was not significantly different, but the number of OTUs of bacteria in samples C1-3, C4-5 and FW was significantly different (*p* < 0.05) (Supplementary Figure 2). OTUs were grouped at the 97% similarity cut off, and diversity indices and richness estimates were calculated for each sample (Supplementary Table 3).

**FIGURE 3:**
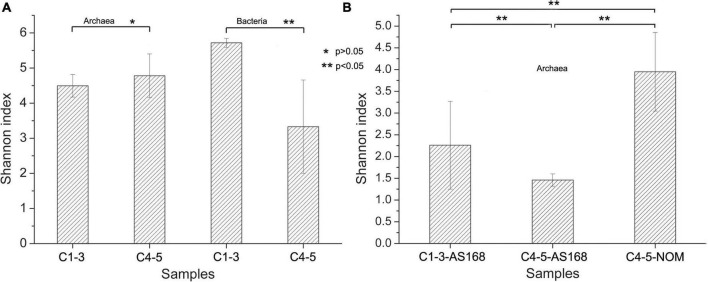
Species diversity of different brine samples revealed by Shannon index. The species diversity was shown using 16S rRNA-based high-throughput sequencing technology under culture-independent **(A)** and culture-dependent approaches **(B)**. *p > 0.05, **p < 0.05. Archaea, archaeal 16S rRNA gene primers were used; Bacteria, bacterial 16S rRNA gene primers were used; AS-168 medium, colonies grown on the AS-168 medium were collected for the archaeal 16S rRNA-based high-throughput sequencing; NOM medium, colonies grown on the NOM medium were collected for the archaeal 16S rRNA-based high-throughput sequencing; C1-3, three samples collected in July 2021; C4-5, two samples collected in August 2018.

The coverage values of all samples exceeded 0.99 (Supplementary Table 3), indicating that the sequencing results can adequately reflect the diversity and structure of microbial community. Similarly, the rarefaction curves for all samples including freshwater samples (FW) were approaching saturation as the sequencing deepens, suggesting sequencing depth was adequate (Figures 2C,D and Supplementary Figures 3a,b). The Chao1, ACE, Simpson and Shannon indices for bacterial communities in samples C1-3 were higher than those in samples C4-5, especially the Shannon index was significantly different (*p* < 0.05) (Supplementary Table 3 and Figure 3A). However, C4 exhibited higher Chao1, ACE and Shannon indices compared to other brine samples when using archaeal primers.

For culture-dependent approach (Cx-168 and Cx-NOM), there was no significant difference in the species diversity (*p* < 0.05) (Supplementary Table 3 and Figure 3B). Interestingly, the bacterial diversity of samples C1-3 was higher than that of Archaea, while it was opposite in samples C4-5.

### Microbial community structure revealed by culture-independent approach

#### Community structure of bacteria and archaea at the phylum, family, and genus level

**FIGURE 4:**
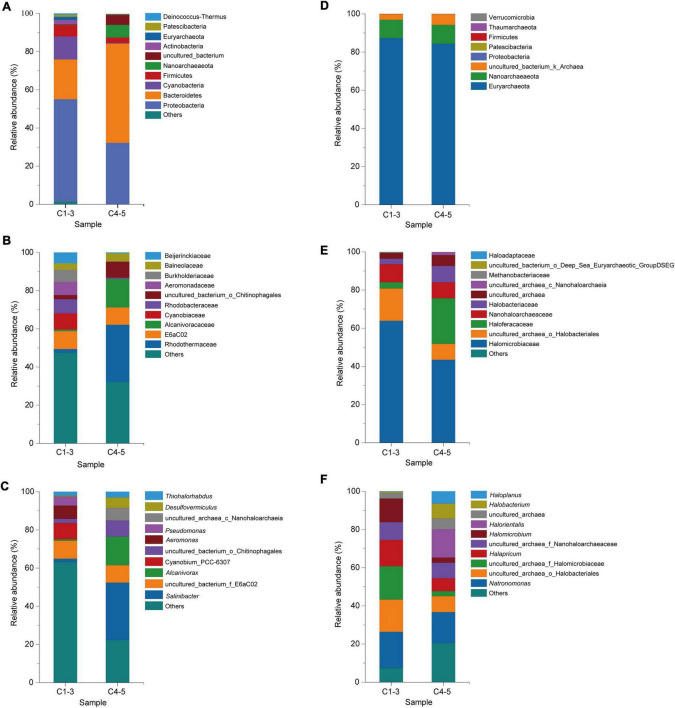
Microbial community of brine samples revealed by 16S rRNA gene sequencing under culture-independent strategy. The community composition of bacteria in brine samples was shown at the phylum **(A)**, family **(B)**, and genus **(C)** levels; while composition of archaea in the brine samples was also shown at the phylum **(D)**, family **(E)**, and genus **(F)** levels; C1-3, three brine samples collected in July 2021; C4-5, two brine samples collected in August 2018.

According to the classification based on the 16S rRNA gene sequence similarity (V3 + V4), a total of 28 bacterial phyla, 218 bacterial families and 345 bacterial genera were identified. The top 10 bacterial classes in different brine samples are vividly exhibited in Figures 4A–C. As shown in Figure 4A, the bacterial community in C1-3 was dominated by phyla Proteobacteria (53.61%), Bacteroidetes (20.85%) and Cyanobacteria (12.04%), followed by Firmicutes (5.90%) and Actinobacteria (2.21%). Coincidentally, the dominant phyla in C4-5 were also Proteobacteria and Bacteroidetes, accounting for 32.22% and 51.99%, respectively. On the other hand, the dominant phyla in freshwater samples (FW) were also Proteobacteria (42.99%), Actinobacteria (27.88%) and Bacteroidetes (12.41%) (Supplementary Figure 4). It’s obvious that although samples C1-3, C4-5 and FW shared the similar dominant phyla, the proportion of them was quite different. For instance, the proportions of phyla Proteobacteria, Cyanobacteria, Firmicutes and Actinobacteria in C4-5 were lower than in C1-3, indicating that their abundance decreased during a long-term indoor static and closed storage. Conversely, the proportion of Bacteroidetes in C4-5 community increased significantly. At the family level, families *E6aC02*, *Cyanobiaceae*, *Rhodobacteraceae*, *Aeromonadaceae*, and *Burkholderiaceae* were the top five dominant taxa in C1-3, but only constituted 38.56% of the total taxa. However, *Rhodothermaceae* (30.01%) was dominant in C4-5 at the family level, followed by families *Alcanivoracaceae* (14.96%) and *E6aC02* (9.10%). Detailed analysis of the bacterial community composition at the genus level revealed that some genera were dominant in C1-3 with a higher proportion, while presenting a much lower proportion in C4-5 or even below detection limit. For example, genera *Cyanobium_PCC-6307* (8.46%), *Aeromonas* (6.91%), and *Pseudomonas* (4.71%) were the dominant genera in C1-3, but almost undetectable in C4-5. Genus *Salinibacter* (30.01%) was the most common in C4-5, followed by genus *Alcanivorax* (14.96%). Apparently, during a long-term indoor storage, genera *Salinibacter*, *Alcanivorax* and *Desulfovermiculus* were flourishing, while genera *Cyanobium_PCC-6307*, *Aeromonas* and *Pseudomonas* were experiencing a recession or even extinction.

Archaeal taxa at the phylum level and family level are shown in Figures 4D,E, respectively. The representative archaeal phyla in C1-3 and C4-5 were Euryarchaeota and Nanoarchaeaeota, accounting for 96.90% and 94.24% of the total OTUs, respectively. At the family level, *Halomicrobiaceae* (63.81%) and *Nanohaloarchaeaceae* (9.45%) were the top dominant families in C1-3. The most abundant family in C4-5 was *Halomicrobiaceae* (43.43%), followed by the family *Nanohaloarchaeaceae* (23.89%). Furthermore, it showed that families *Haloferacaceae* and *Halobacteriaceae* were more abundant in C4-5 than C1-3, while families *Halomicrobiaceae* and *Nanohaloarchaeaceae* were the opposite. To better explain the structure of the archaeal community in different brines, the relative abundance and classification of OTUs were analyzed at the genus level (Figure 4F). Genera *Natronomonas* (18.89%), *Halapricum* (13.73%) and *Halomicrobium* (12.35%) were the dominant genera in C1-3. In C4-5, the top dominant taxa were genera *Natronomonas* (16.18%), *Halorientalis* (14.80%), *Halobacterium* (7.82%), *Haloplanus* (6.46%) and *Halomicrobium* (2.67%). It was found that the abundance of genera *Halapricum* and *Halomicrobium* decreased sharply after a long-term indoor storage, while genera *Halorientalis*, *Halobacterium*, and *Haloplanus* became dominant.

#### Community composition of haloarchaea at the genus level revealed by limited clone sequencing

**FIGURE 5:**
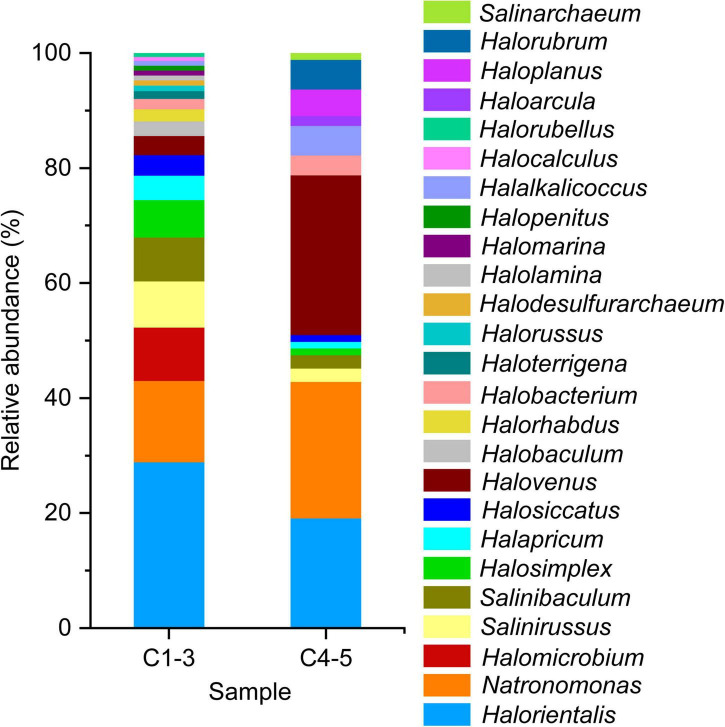
Haloarchaeal composition in brine samples revealed by clone library at the genus level under culture-independent approach. Almost complete length of the 16S rRNA gene was obtained by using F8 and R1462 primer pair. C1-3, three brine samples collected in July 2021; C4-5, two brine samples collected in August 2018.

The limited clone library strategy sequenced 185 clones selected randomly from samples C1, C2, C3, C4, and C5. These 185 sequenced 16S rRNA gene sequences belonged to 25 genera (Figure 5). Among them, genera *Halorientalis* (29.83%), *Salinirussus* (16.66%), *Natronomonas* (14.16%) and *Halomicrobium* (9.24%) were the dominant groups with a relative higher proportion in C1-3. In C4-5, genus *Halovenus* (28.75%) accounted for the highest proportion, followed by genera *Halorientalis* (21.79%), *Natronomonas* (21.07%), and *Haloplanus* (8.39%). Intriguingly, genus *Halomicrobium* accounting for 9.24% in C1-3 was not detected in C4-5. However, genus *Haloplanus* accounting for 8.39% in C4-5 was not detected in C1-3. Thus, the patterns of change for genera *Halomicrobium* and *Haloplanus* were the opposite.

Sequence similarity search of these cloned 16S rRNA gene sequences against public database using Basic Local Alignment Search Tool (BLAST; see text footnote 2) revealed that the majority of these cloned sequences showed a relatively lower sequence identity (<95%). It supposed that there were a large number of potential new species or genera in hypersaline environments. Meanwhile, these results also indicated that high through-put sequencing and cloning library were both culture-independent methods showing a significant difference in revealing microbial community structure.

### Microbial community structure uncovered by culture-dependent methods

**FIGURE 6:**
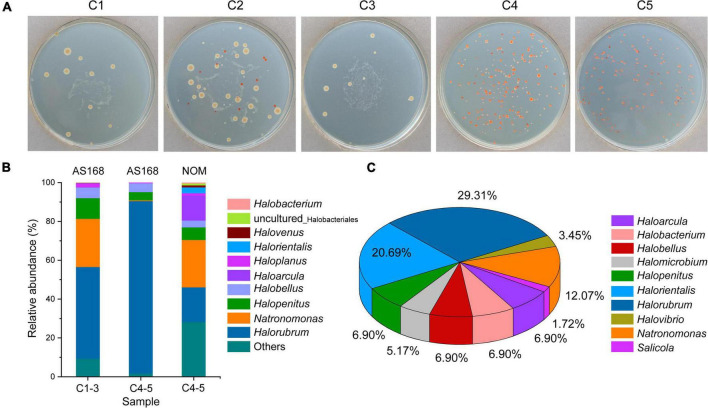
Halophilic archaea and halotolerant bacteria isolated from brine samples. Cultivation of halophilic microbes from brine samples using the AS-168 agar plates **(A)**. Cultures grown on the AS-168 and NOM agar plates were collected by washing with 20% (w/v) sterilized NaCl solution for archaeal 16S rRNA gene (V3 + V4) high-throughput sequencing **(B)**. Microbial composition of randomly isolated strains identified by sequencing the almost complete 16S rRNA gene combined with sequence similarity search **(C)**. C1-3, three brine samples including C1, C2, and C3 collected in July 2021; C4-5, two brine samples including C4 and C5 collected in August 2018.

Halophilic microbes in different brine samples were cultivated by using AS-168 and NOM media, respectively. On AS-168 agar plates, it was evident that the number and species of halophilic microorganisms from C4-5 exceeded those from C1-3 (Figure 6A). The chocolate-colored with white surrounding colonies cultivated from C1-3 belonged to the genus *Salicola*, which were completely absent in C4-5 on AS-168 agar plates.

To gain more information, colonies grown on AS-168 and NOM agar plates were washed with 20% (w/v) sterilized NaCl solution and then collected for high-throughput sequencing using archaeal 16S rRNA gene primers. The top 10 archaeal genera detected in different medium are shown in Figure 6B. Genera *Halorubrum* (47.23%), *Natronomonas* (24.78%) and *Halopenitus* (10.73%) were the dominant Haloarchaea in C1-3-AS168. Similarly, genus *Halorubrum* (88.69%) was also determined to be the most dominant group in C4-5-AS168, followed by genera *Halobellus* (4.56%) and *Halopenitus* (4.25%). Although genus *Halorubrum* showed the highest richness in both C1-3-AS168 and C4-5-AS168, the proportion varied from different sample sets. Genera *Natronomonas* (24.37%), *Halorubrum* (17.91%) and *Haloarcula* (12.81%) were the dominant genera in C4-5-NOM. Among them, *Natronomonas* and *Haloarcula* were more abundant in C4-5-NOM than C4-5-AS168, indicating that NOM medium was more suitable for their growth. Compared to NOM medium, AS-168 was a eutrophic environment. It reflected that as a typical chemoheterotrophic halophilic archaeon, *Halorubrum* spp. may prefer a eutrophic environment. Generally, oligotrophic environments were more suitable for the isolation of more different halophilic archaea (Figure 6B).

Through cultivation and a series of streaking, 62 halophilic microorganisms including 12 possible new species (sequence identity < 97.5%, see “Data availability statement”) were isolated from different brine samples by using AS-168 and NOM media. Next, they were classified into 10 genera such as *Halorubrum*, *Halorientalis*, *Natronomonas*, and *Halovibrio* based on the 16S rRNA gene sequence similarity search (Figure 6C).

### Microbial co-occurrence network analyses

**FIGURE 7:**
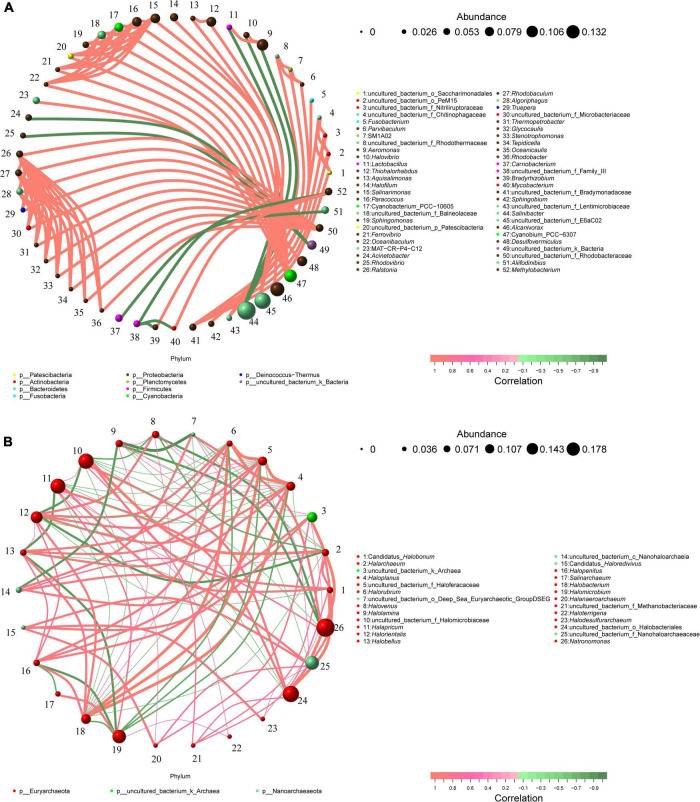
Co-occurrence networks of microbial communities in different brine samples based on correlation analysis. The nodes in network are colored by phylum. Co-occurrence networks of bacterial communities **(A)** and archaea communities **(B)**; The connections indicate strong spearman’s (ρ > 0.7) and significant (*p* < 0.05) correlations. The size of each node is proportional to the relative abundance of specific genus.

The bacterial genera and archaea genera with a relative abundance more than 0.1% were selected as study objects, and the potential interactions of these taxa were analyzed. Co-occurrence network taxa were highly significant network hubs (ρ > 0.7, *p* < 0.05; Figures 7A,B). The bacterial network consisted of 52 nodes and 100 edges, and the average degree and the clustering coefficient were 3.85 and 1.29, respectively. In the bacterial network, most of the correlations were positive (positive correlation ratio: 91%, Figure 7A). The nodes in the bacterial network were divided into 10 bacterial phyla. Among them, Proteobacteria accounted for 53.85% of all nodes, showing a strong intra-phylum correlation. However, members of Actinobacteria and Bacteroidetes showed more positive correlations with other bacterial genera, especially with Proteobacteria. The top nine keystone genera with the highest number of connections in the bacterial network were *Ralstonia*, *Rhodobaculum*, *uncultured_bacterium_f_Rhodobacteraceae*, *Salinarimonas*, *Paracoccus*, *Cyanobacterium_PCC-10605*, *uncultured_bacterium_f_Balneolaceae*, *Algoriphagus*, and *Cyanobium_PCC-6307*.

The archaeal network was composed of 26 nodes and 100 edges, and the average degree and clustering coefficient were 7.69 and 0.61, respectively (Figure 7B). Among the only three Archaeal phyla, the Euryarchaeota was the most dominant, accounting for 80.76% of all nodes. The next was Nanoarchaeaeota (15.38%), which had the highest number of associations with Euryarchaeota. The top five genera with the most connections in the archaeal network were *Haloplanus*, *uncultured_bacterium_f_Halofracaceae*, *Halobacterium*, *Halapricum*, and *Halorientalis*.

## Discussion

The Dingyuan Salt Mine in Anhui Province, located in central China, is endowed with the characteristics of continental basin deposits, whose salt-bearing layer was formed in the Early Tertiary Oligocene and is dominated by river-lake facies deposition (Supplementary Figures 1a,b; Chen et al., 2019). At present, water dissolution (fresh water-in and brine-out) is the main method for salt mining (Figures 1A,B). In detail, the mining process involves injecting fresh water (solvent) into the deposit to dissolve salt minerals *in situ* into a flowing solution (brine), and then the brine coming out (Wang, 1999). In this study, the composition of microbial communities in brines after long-term indoor static and closed storage (C4-5) and in freshly collected brines (C1-3) was preliminarily compared by a combination of culture-independent and culture-dependent methods. Environmental conditions such as high salinity, hypoxia, and slightly alkaline pH are driving factors shaping microbial community structure and forming corresponding adaptability after long-term indoor sealed storage in this study.

It is widely acknowledged that salinity is an important factor affecting the richness and diversity of bacteria and archaea (Kalwasinska et al., 2017; Banda et al., 2021). The DNA concentration of freshwater samples (FW) used to dissolve the rock salt buried underground in this study is significantly higher than that of brine samples (C1-5) (*p* < 0.05; Supplementary Table 2 and Figure 2A), illustrating that microorganisms in freshwater declined steeply or even disappeared after entering a hypersaline environment. Besides, it also means that environmental filtering prevents the efficient colonization and persistence of non-tolerant species in hypersaline environments (Triado-Margarit et al., 2019). It is worth noting that the bacterial diversity of samples C1-3 is higher than that of archaea, which is in the opposite manner for samples C4-5 (Supplementary Table 3 and Figure 3A). And the diversity of bacterial community in samples C1-3 is significantly higher than that in samples C4-5 (*p* < 0.05; Supplementary Table 3 and Figure 3A). It is worth noting that some of the error bars in Figures 2A, 3A,B vary greatly, which may be caused by the small sample size. However, the tendency reflected by the differences between these samples (C1-3 and C4-5) are still obvious and persuasive. The result showed that the diversity of bacterial communities in the brine samples decreased significantly after long-term indoor sealed storage. At the phylum level, long-term indoor sealed storage profoundly altered bacterial community structure, i.e., the relative abundance of some bacterial communities varied much more than archaeal communities (Figures 4A,D). At the same time, studies have shown that archaea can withstand environmental stress better than bacteria, and obtain a stable community structure within a certain time-frame (de León-Lorenzana et al., 2017; Mani et al., 2020). Therefore, we believe that archaea in brine samples are more adaptive than bacteria in the hypersaline environment under a long-term indoor sealed storage condition.

In this paper, bacterial and archaeal communities in different brine samples were analyzed by Illumina high-throughput sequencing technology. Overall, the bacterial communities in the brine samples are mainly composed of Proteobacteria and Bacteroidetes (Figure 4A), which is consistent with other researches (Boujelben et al., 2012; Kalwasinska et al., 2018; Mazière et al., 2021). In fact, Proteobacteria and Bacteroidetes are also the dominant phyla in other saline waters (Boujelben et al., 2012; Kalwasinska et al., 2018; Cardoso et al., 2019), playing an important role in carbon and nitrogen cycling (Bernhard et al., 2012; Wong et al., 2015). The result also shows that these brine samples have the characteristics of dominant bacterial taxa commonly found in other hypersaline environments.

Further analysis reveals that although samples C1-3 and C4-5 shared the same dominant bacterial phyla, their relative abundances are quite different (Figure 4A). As it shows in the result that the abundance of Proteobacteria may decrease greatly after long-term indoor sealed storage, whereas the abundance of Bacteroidetes is the opposite. Similarly, genera *Salinibacter* and *Alcanivorax* are dominant in C4-5, but less abundant in C1-3 (Figure 4C). *Cyanobium_PCC-6307*, *Aeromonas* and *Pseudomonas* are the dominant genera in C1-3, but are barely detectable in C4-5 (Figure 4C). Among them, genus *Salinibacter* is an extreme halophilic genus with archaeal properties in Bacteroidetes, also an important part of bacterial communities in various high-salt environments (Bachran et al., 2019; Perez-Fernandez et al., 2019; Cycil et al., 2020). *Salinibacter* spp. generally, requires light-driven pumps for growth and maintenance of ion gradients across the cell membrane (Doğan and Kocabaş, 2021). The low levels of genus *Salinibacter* in C1-3 may be attributed to relatively lower levels of solar radiation and lower temperatures (Kalwasinska et al., 2018). Meanwhile, the enrichment of genus *Salinibacter* is also one of the reasons for the largest proportion of Bacteroidetes in C4-5.

Compared with bacterial taxa, fewer Archaeal phyla can be found in these samples, with most of the sequences belonging to Euryarchaeota, followed by Nanoarchaeaeota (Figure 4D). And the relative abundance difference of the dominant phyla in samples C1-3 and C4-5 is small (Figure 4D). However, the opposite is true at the genus level. The relative abundance of genera *Halapricum* and *Halomicrobium* in samples C1-3 is much higher than those in samples C4-5, while the relative abundance of genera *Halorientalis*, *Halobacterium* and *Haloplanus* in samples C4-5 is much higher than those in samples C1-3 (Figure 4F). Interestingly, genus *Natronomonas* dominates in all samples (Figure 4F). As observed elsewhere, genus *Natronomonas* is one of the most successful ecological taxa able to survive in hypersaline environments (Chen et al., 2020; Banda et al., 2021; Satari et al., 2021; Zhu et al., 2021).

It should not be ignored that species in genera *Alcanivorax* and *Halorientalis* were blooming in hypersaline environment in long-term indoor storage, becoming the dominant genera (Figures 4C,F). Genus *Alcanivorax* is a ubiquitous marine hydrocarbonoclastic genus, which dominates in many oil-contaminated environments (Scoma and Boon, 2016). Known for its preference for metabolizing hydrocarbons and crude oil derivatives (Zadjelovic et al., 2020). And the members of genus *Halorientalis* were also reported to be capable of degrading hydrocarbons (Khalid et al., 2021). It is worth noting that the formation process of rock salt and petroleum is always closely related. Petroleum consisting of hydrocarbons with different carbon chain length is covered by a thick stratum of salt. Hydrocarbons with a relatively low density can leak into the upper salt stratum through cracks created by geological movements (Sonnenfeld and Perthuisot, 1984). Additionally, previous studies have shown that the Hefei Basin (Anhui province, China), where the Dingyuan Salt Mine is located, exhibits ideal geological conditions for petroleum formation (Jia et al., 2001; Dai et al., 2011). The brine sample smelled of petrol. Therefore, the brine samples are likely to contain petroleum and hydrocarbons, and such favorable environmental conditions may lead to the enrichment of genera *Alcanivorax* and *Halorientalis*. Hydrocarbons degrading microorganisms were enriched in the presence of favorable substrates during a relative long period of storage.

Clone library technology has been widely used to study microbial communities in different habitats (Jones et al., 2009; Xiao et al., 2013; Wang et al., 2018; Chen et al., 2020), which is also applied in this study. It can be found that the dominant genus with the highest proportion in C1-3 is genus *Halorientalis*, while in C4-5 it is genus *Halovenus* (Figure 5), which is inconsistent with the high-throughput sequencing results. Previous studies have also shown that the diversity of microbial communities can fluctuate severely along with the size of the clone library (Li et al., 2017). Therefore, it is normal to get different results between the two methods.

With the development of molecular methods, culture-independent methods are considered more effective than culture-dependent methods (Dakal and Arora, 2012), because the latter can only detect 1–5% of all microorganisms in the sample (Ma et al., 2015). Nevertheless, the methods of culture-dependent are still an indispensable technique to acquire microbial species with tremendous application potential and to understand their ecophysiological and environmental functions (Vandamme et al., 1996; Sfanos et al., 2005). In this study, genera *Halorubrum*, *Halobellus*, and *Halopenitus* are the dominant taxa in C4-5-AS168, but genera *Natronomonas*, *Halorubrum*, and *Haloarcula* have a higher proportion in C4-5-NOM (Figure 6B). Among them, the relative abundance of genus *Halorubrum* in C4-5-AS168 is nearly five times that in C4-5-NOM, indicating that the eutrophic environment of AS-168 may make it more competitive.

Furthermore, a large proportion of microbial species are uncultured, which tends to make some opportunistic species predominant in isolates. Therefore, culture-dependent approaches introduce numerous biases, and generally do not select the most abundant taxa in the environment. Rather, they select the microorganisms that grow best under the culture conditions used. The results often differ from the actual distribution of microbial taxa in the environment (Henriet et al., 2014; Naghoni et al., 2017). In this study, a total of 62 halophilic microorganisms including 12 possible new species (sequence identity < 97.5%) isolated from the two media belong to 10 genera (Figure 6C). Notably, their sequence similarity search based on 16S rRNA gene sequences all show relatively higher sequence identity (>95%). However, most of the 16S rRNA gene sequences obtained by clone library approach exhibit a lower sequence identity (<95%). The unpredictable alteration of template may happen in different cycles during the PCR amplification in clone library construction, which tends to form a larger number of chimeras. Therefore, clone library approach based on PCR amplification of total environmental DNA may severely overestimate the species diversity (Stevens et al., 2013).

Co-occurrence networks can reveal interactions between different taxa in microbial communities, which can be competitive or cooperative (Faust and Raes, 2012; He et al., 2019). In this study, the positive correlation among bacterial networks is 91% (Figure 7A), while that among archaeal networks is only 64% (Figure 7B). This suggests that bacterial communities in brine samples are more likely than archaeal communities to survive in harsh environments through synergies. In addition, genera *Ralstonia*, *Rhodobaculum*, *uncultured_bacterium_f_Rhodobacteraceae*, *Salinarimonas*, *Paracoccus*, *Cyanobacterium_PCC-10605*, *uncultured_bacterium_f_Balneolaceae*, *Algoriphagus* and *Cyanobium_PCC-6307* are found to be highly associated taxa in the bacterial network. Similarly, genera *Haloplanus*, *uncultured_bacterium_f_Halofracaceae*, *Halobacterium*, *Halapricum* and *Halorientalis* are more connected in the archaeal network. These microbial taxa are considered as keystone taxa due to their highly connected nodes (Hu et al., 2018; Guo et al., 2019). Compared with other taxa in the network, these taxa may play a vital part in maintaining the stability of ecological network structure and function (Faust and Raes, 2012; Shi et al., 2019).

Interestingly, the average relative abundances of keystone taxa (except genus *Cyanobium_PCC-6307*) in the bacterial network were fairly low (0.81%∼2.51%). The results indicated the significance of low-abundance genera in bacterial communities. Although the abundance of such genera may not be high, more attention should be paid to them being the key nodes in bacterial communities (Guo et al., 2019). However, the average relative abundances of keystone genera (except *uncultured_bacterium_f_Halofracaceae*) in archaeal network ranged from 2.71% to 10.95%. The result of archaea is different from Bacteria in network. The dominant archaea are also key nodes in archaeal network. These differences may be attributed to the different adaptation mechanisms of bacteria and archaea, which are beneficial to their survival and development of their respective taxa. On the other hand, members of the genera *Salinarimonas* and *Paracoccus* were previously isolated in oil-contaminated environments, indicating their role in hydrocarbon bioremediation (Zhang et al., 2020; Procópio, 2021). And bacteria belonging to *Algoriphagus* were also confirmed as an oil-degrading bacterium (Wang et al., 2014). Meanwhile, members of the genera *Halobacterium* and *Halorientalis* were reported to be capable of degrading hydrocarbons (Kumar et al., 2020). Therefore, these keystone taxa may play important roles in ecological processes, especially in the remediation of oil-polluted hypersaline environments.

## Conclusion

In this work, the microorganisms in the brine of salt mine were studied in details for the first time by combining culture-independent and culture-dependent methods. Our conclusions are:

(1) After long-term indoor airtight storage, the species diversity of bacterial communities in brine samples decreased significantly (*p* < 0.05), while that of archaeal communities did not change significantly. The composition of the dominant bacterial and archaeal phyla in different samples was similar, but their relative abundances of dominant phyla are significantly different. Among them, halotolerant genera *Salinibacter* and *Natronomonas* were the predominant inhabitants in brine samples, suggesting that they play a crucial role in this environment.

(2) A total number of 62 halophilic microorganisms including 12 possible new species (sequence identity < 97.5%) belong to 10 genera were isolated from brine of inland salt mine through culture-dependent method before these inhibiting species went extinct with salt mining. Extremophiles from hypersaline environment are of great significance in special biotechnological applications, and in understanding their ecophysiological and environmental functions.

(3) Network analysis showed that the bacteria in brine samples were more likely than the archaea to survive in harsh environments through synergies. Keystone taxa with highly connected nodes (such as genera *Ralstonia*, *Rhodobaculum*, *Haloplanus*, etc.) play an important role in maintaining the stability of ecological network structure and function.

(4) The brine samples are likely to contain petroleum and hydrocarbons, and such favorable environmental conditions led to the enrichment of specific genera *Alcanivorax* and *Halorientalis*, which are capable of degrading hydrocarbons. In addition, the genera *Salinarimonas*, *Paracoccus*, *Algoriphagus* and *Halobacterium* in keystone taxa also have this ability. The interesting phenomenon reflects that these taxa may have significant contributions in the bioremediation of oil-contaminated hypersaline environments.

Overall, the results of this study will expand the understanding of microbial diversity in extreme environments. On the other hand, it is conducive to explore the functional roles or environmental niches inhabited by various microorganisms in extreme environments.

## Data availability statement

The datasets presented in this study can be found in online repositories. The names of the repository/repositories and accession number(s) can be found below (check Supplementary Table 4 for details): NCBI -

(1) PRJNA787045 (https://submit.ncbi.nlm.nih.gov/subs/sra/SUB10779502/overview);

(2) PRJNA791781 (https://submit.ncbi.nlm.nih.gov/subs/sra/SUB10837144/overview);

(3) PRJNA787012 (https://submit.ncbi.nlm.nih.gov/subs/sra/SUB10721350/overview);

(4) PRJNA787052 (https://submit.ncbi.nlm.nih.gov/subs/sra/SUB10779714/overview);

(5) OM184316-OM184500 (https://submit.ncbi.nlm.nih.gov/subs/genbank/SUB10907784/overview);

(6) OL979230-OL979291 (Possible new species: OL979250, OL979251, OL979252, OL979254, OL979260, OL979266, OL979267, OL979268, OL979269, OL979280, OL979282, and OL979283) (https://submit.ncbi.nlm.nih.gov/subs/genbank/SUB10837125/overview).

## Author contributions

SC and JH: conceptualization and funding acquisition. SC, SS, DT, JK, YL, and TH: data curation. SC, SS, and DT: investigation. SC and DT: methodology. SC, DT, and JK: writing – original draft. SC, JH, DT, and JK: writing – review and editing. All authors have read and agreed to the published version of the manuscript.

## Footnotes

1
https://desktop.arcgis.com/en/

2
https://blast.ncbi.nlm.nih.gov/Blast.cgi
