# Supplementary material for: Microbial community structure and shift pattern of industry brine after a long-term static storage in closed tank
Source: Front Microbiol. 2022 Sep 2;13:975271. doi: 10.3389/fmicb.2022.975271 (PMC9478951; doi:10.3389/fmicb.2022.975271)
Supplement: Supplementary file 1 [file Data_Sheet_1.pdf]

Supplementary materials

Table S1. Primers used in this study.

Name	Sequence (5'-3')
F8	TTGATCCTGCCGGAGGCCATTG
R1462	ATCCAGCCGCAGATTCCCCTAC
27F	AGAGTTTGATCMTGGCT
1492R	CGGTTACCTTGTTACGACTT
338F	ACTCCTACGGGAGGCAGCA
806R	GGACTACHVGGGTWTCTAAT
Arch349F	GYGCASCAGKCGMGAAW
Arch806R	GGACTACVSGGGTATCTAAT

Table S2. Total DNA isolated from the brines.

**Sample**	C1[a]	C2[a]	C3[a]	C4[b]	C5[b]	ddH_2O	Air
**DNA amount (ng)**	660.00	784.50	939.78	2975.28	1921.02	58.98/62.83	88.98/75.32
**Mean±STDEV**	794.76±140.17			2448.15±745.47		60.91±2.72	82.15±9.66

Note: a, Brine collected in July, 2021; b, Brine collected in August, 2018.

Table S3. Species diversity of Archaea and Bacteria in brine uncovered by culture-dependent and culture-independent approaches.

Method	Group	Sample	ACE	Chao1	Simpson	Shannon	Coverage
Culture-independent method	Species diversity of Archaea	C1[a]	156.69	156.00	0.93	4.73	0.9999
		C2[a]	174.44	171.00	0.93	4.62	0.9997
		C3[a]	159.49	163.43	0.90	4.13	0.9997
		C4[b]	191.16	190.86	0.94	5.22	0.9998
		C5[b]	155.10	144.14	0.90	4.34	0.9997
	Species diversity of Bacteria	C1[a]	474.65	478.63	0.93	5.58	0.9996
		C2[a]	441.33	447.69	0.96	5.83	0.9994
		C3[a]	425.10	427.06	0.95	5.74	0.9998
		C4[b]	313.56	325.95	0.91	4.27	0.9995
		C5[b]	251.75	259.50	0.65	2.39	0.9996
Culture-dependent method and Species diversity of Archaea	in AS-168 medium	C1[a]	131.69	107.00	0.83	3.26	0.9995
		C2[a]	187.00	186.93	0.22	1.24	0.9996
		C3[a]	206.61	213.00	0.50	2.28	0.9997
		C4[b]	152.50	156.63	0.40	1.56	0.9994
		C5[b]	137.49	138.40	0.51	1.36	0.9994
	in NOM medium	C4[b]	130.51	128.46	0.84	3.31	0.9993
		C5[b]	264.62	270.00	0.79	4.59	0.9998

Note: a, Brine collected in July, 2021; b, Brine collected in August, 2018.

Table S4. The datasets generated and analyzed in the current study.

Methods	Samples	BioProject IDs	Links
The high-throughput DNA sequencing of bacteria from environmental DNA	C1[a], C2[a], C3[a], C4[b], C5[b]	PRJNA787045	https://submit.ncbi.nlm.nih.gov/subs/sra/SUB10779502/overview
	JSH	PRJNA791781	https://submit.ncbi.nlm.nih.gov/subs/sra/SUB10837144/overview
The high-throughput DNA sequencing of archaea from environmental DNA	C1[a], C2[a], C3[a], C4[b], C5[b]	PRJNA787012	https://submit.ncbi.nlm.nih.gov/subs/sra/SUB10721350/overview
The high-throughput DNA sequencing of archaea after cultivation	C1[a], C2[a], C3[a], C4[b], C5[b] (AS-168 medium) and C4[b] and C5[b] (NOM medium)	PRJNA787052	https://submit.ncbi.nlm.nih.gov/subs/sra/SUB10779714/overview
The clone library of archaea from environmental DNA	C1[a], C2[a], C3[a], C4[b], C5[b]	OM184316-OM184500	https://submit.ncbi.nlm.nih.gov/subs/genbank/SUB10907784/overview
Pure cultures isolated after cultivation by AS-168 and NOM medium	C1[a], C2[a], C3[a], C4[b], C5[b]	OL979230-OL979291	https://submit.ncbi.nlm.nih.gov/subs/genbank/SUB10837125/overview

Note: a, Brine collected in July, 2021; b, Brine collected in August, 2018.

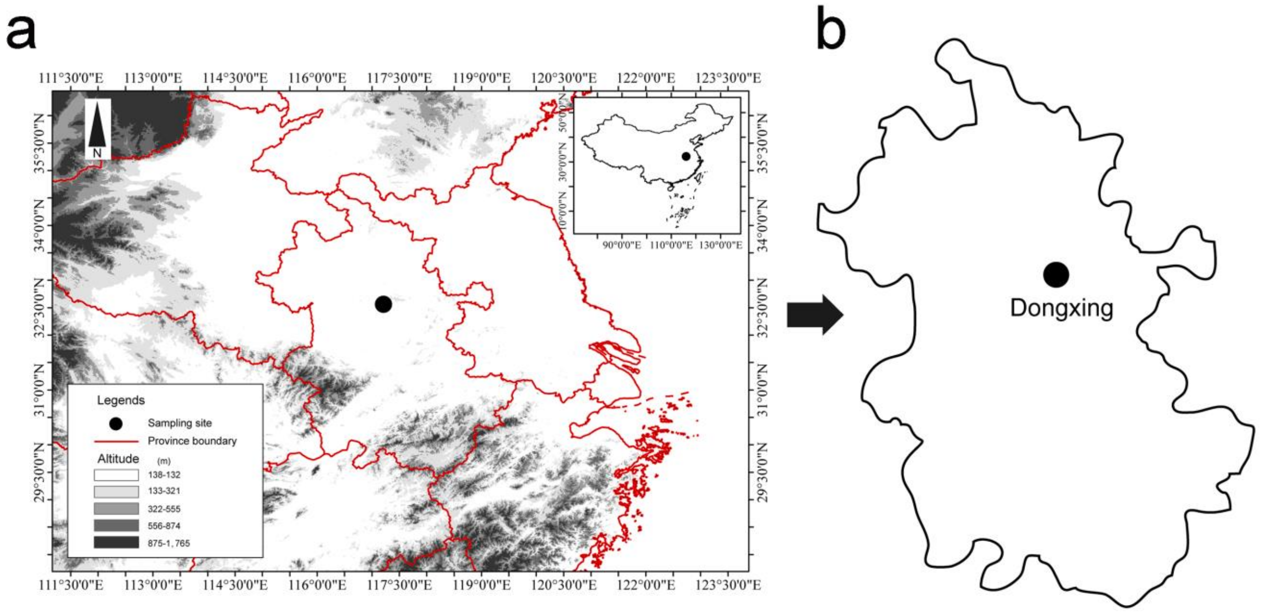

Figure S1. Location of the sampling site. (a) Geographic location of the sampling site (filled circle) was generated with ArcGIS 10.2 sofware (https://desktop.arcgis.com/en/). Different shades of gray shows different altitudes. Sampling site on the map of China is shown on the top right corner. Longitude (top and bottom) and latitude (left and right), and province boundary (red line) are also shown. Samples were collected from Dongxing Town, Dingyuan County, Anhui Province in the east of China. (b) The amplified map of the Anhui Province shows Dongxing Town where the samples were collected.

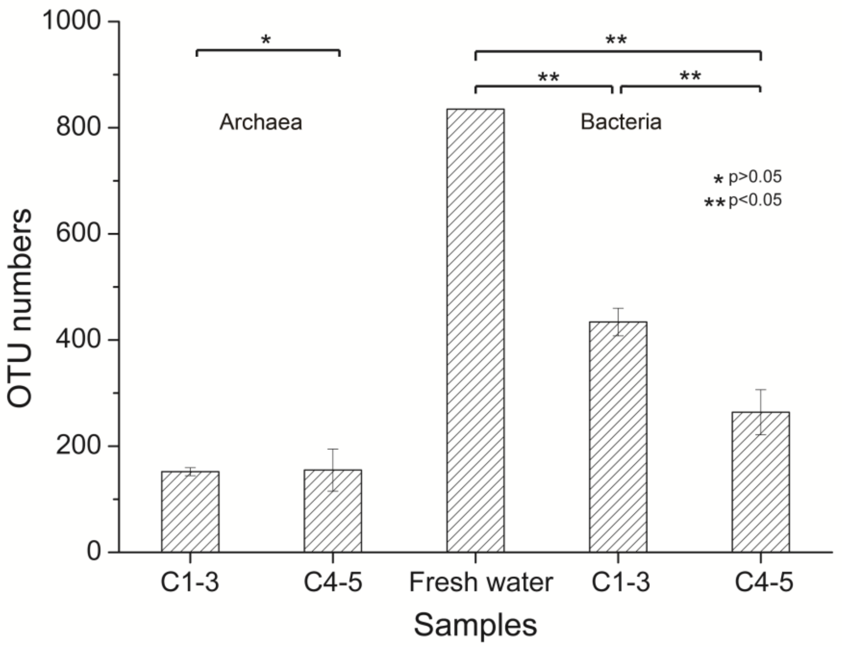

Figure S2. Comparison of archaeal and bacterial OTU numbers in brines collected in 2021 and 2018 through 16S rRNA amplicon sequencing. C1-3, three brine samples collected in 2021; C4-5, two brine samples collected in 2018; Fresh water, the natural water used for brine production.

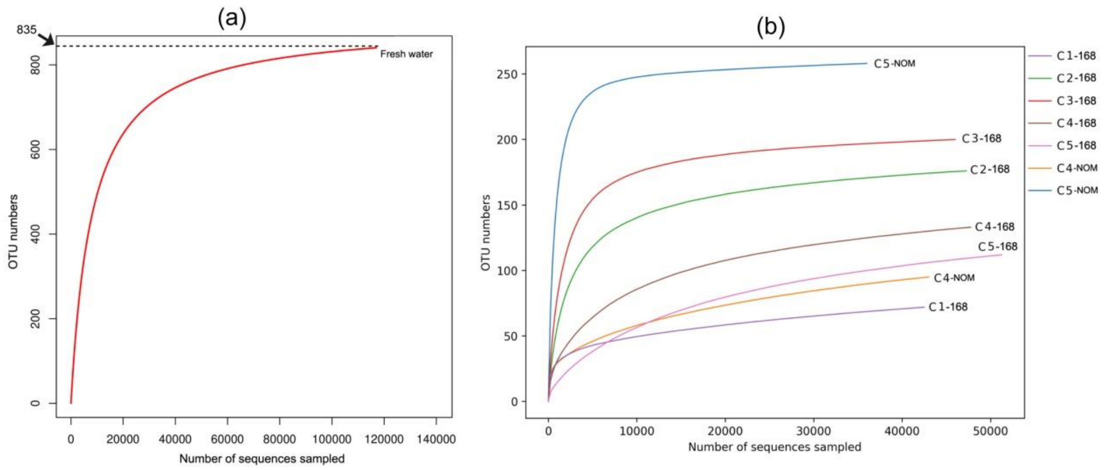

Figure S3. Rarefaction curve. Bacterial 16S RNA amplicon sequencing of the fresh water (a). Archaeal 16S RNA amplicon sequencing of cells grown on the AS-168 medium and NOM medium; C1-C3, brine samples collected in 2021; C4-C5, brine samples collected in 2018; Cx-168, culture-dependent approach was performed on AS-168 medium; Cx-NOM, culture-dependent approach was performed on NOM medium (b).

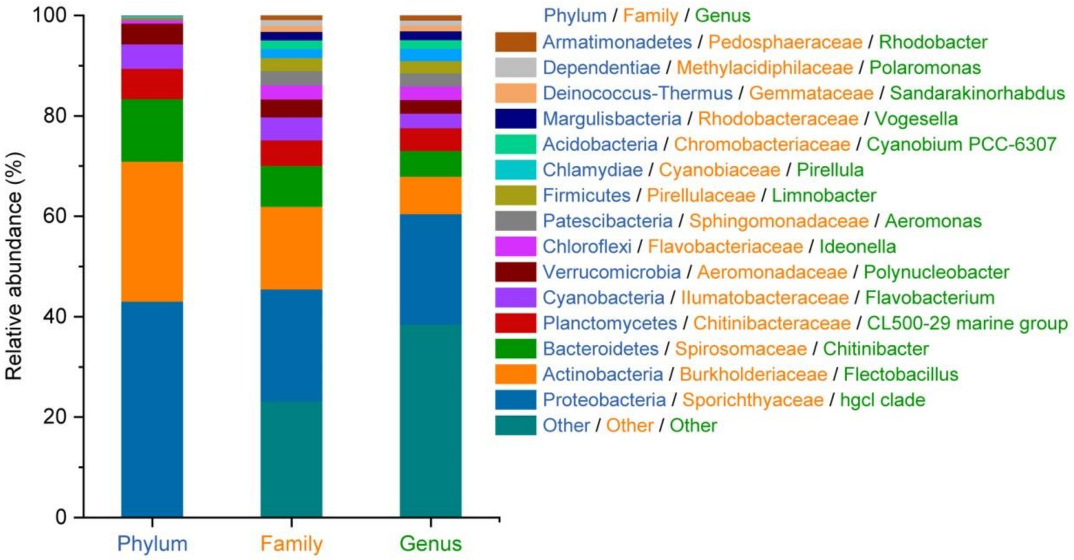

Figure S4. Community composition of bacteria in fresh water at the phylum (blue), family (orange) and genus (green) level.
